# ZP2495 Protects against Myocardial Ischemia/Reperfusion Injury in Diabetic Mice through Improvement of Cardiac Metabolism and Mitochondrial Function: The Possible Involvement of AMPK-FoxO3a Signal Pathway

**DOI:** 10.1155/2018/6451902

**Published:** 2018-01-17

**Authors:** Shuang Li, Hao Wu, Dong Han, Mingming Zhang, Na Li, Weihua Yu, Dongdong Sun, Zhongchan Sun, Sai Ma, Erhe Gao, Congye Li, Min Shen, Feng Cao

**Affiliations:** ^1^Department of Cardiology & National Clinical Research Center of Geriatrics Disease, Chinese PLA General Hospital, Beijing 100853, China; ^2^Department of Cardiology, Chengdu Military General Hospital, Chengdu 610083, China; ^3^Department of Toxicology, School of Public Health, Fourth Military Medical University, Xi'an, Shaanxi 710032, China; ^4^Department of Cardiology, Xijing Hospital, Fourth Military Medical University, Xi'an, Shaanxi 710032, China; ^5^Department of Cardiology, Tangdu Hospital, Fourth Military Medical University, Xi'an, Shaanxi 710032, China; ^6^Center of Translational Medicine, Temple University School of Medicine, Philadelphia, PA 19107, USA

## Abstract

Coronary heart disease patients with type 2 diabetes were subject to higher vulnerability for cardiac ischemia-reperfusion (I/R) injury. This study was designed to evaluate the impact of ZP2495 (a glucagon-GLP-1 dual-agonist) on cardiac function and energy metabolism after myocardial I/R injury in db/db mice with a focus on mitochondrial function. C57BLKS/J-lepr^+^/lepr^+^ (BKS) and db/db mice received 4-week treatment of glucagon, ZP131 (GLP-1 receptor agonist), or ZP2495, followed by cardiac I/R injury. The results showed that cardiac function, cardiac glucose metabolism, cardiomyocyte apoptosis, cardiac mitochondrial morphology, and energetic transition were improved or ameliorated by ZP2495 to a greater extent than that of glucagon and ZP131. *In vitro* study showed that ZP2495, rather than glucagon, alleviated mitochondrial depolarization, cytochrome C release, and mitochondria ROS generation in neonatal rat ventricular myocytes subjected to high-glucose and simulated I/R injury conditions, the effects of which were weaker in the ZP131 group. Furthermore, the expressions of Akt, FoxO3a, and AMPK phosphorylation were elevated by ZP2495 to a greater extent than that of ZP131. In conclusion, ZP2495 may contribute to the improvement of cardiac function and energy metabolism in db/db mice after myocardial I/R injury by improving mitochondrial function possibly through Akt/FoxO3a and AMPK/FoxO3a signal pathways.

## 1. Introduction

Type 2 diabetes is one of the strong independent risk factors for cardiovascular disease and death [1, 2]. Lots of studies including our previous studies have demonstrated that patients with type 2 diabetes have higher vulnerability of cardiac ischemia/reperfusion (I/R) injury as a result of the exposure to abnormal substrate and cytokines [3, 4]. However, effective strategies which can reduce cardiac I/R injury under diabetic conditions are not well developed in a clinic setting.

Glucagon-like peptide-1 (GLP-1) is derived from a proglucagon precursor and secreted by intestinal L-cells in response to oral nutrient ingestion, which acts through G protein-coupled receptor (GLP-1R) on pancreatic beta-cells to exert glucoregulatory and insulinotropic actions [5, 6]. GLP-1R agonists have been reported to have cardiac and vascular actions in rodents and humans, including effects on myocardial contractility, blood pressure, cardiac output, and cardioprotection [7–9]. Glucagon exerts biochemical and physiological effects on heart muscle partly by stimulating Ca^2+^ currents via cAMP production and inhibition of phosphodiesterases [9–11], leading to increased contractility. Although cardiac contractility can be enhanced by glucagon administration, it will increase cardiac mortality since glucagon increases cardiomyocyte consumption of energy after myocardial infarction [12]. Therefore, it will be ideal to find a medicine with both inotropic and metabolic effects that could potentially have beneficial effects on the prevention of the heart injury induced by ischemia.

Earlier reports suggested the combination of GLP-1 and glucagon makes an attractive proposition for obesity therapy [13, 14]. However, the protective role of glucagon and GLP-1 dual-agonist against I/R injury has not previously been demonstrated. Even in the absence of diabetes, a switch from glucose to fatty acid metabolism contributes to the severity of an ischemic injury and can impair functional recovery during and following ischemia. This is particularly evident during the reperfusion phase of the ischemic myocardium [15–17]. As a result, diabetes-induced changes in energy metabolism have the potential to significantly impact on the ability of the heart to withstand an I/R injury [18]. This study was designed to evaluate the impact of a glucagon-GLP-1 dual-agonist ZP2495 on cardiac function and energy metabolism after myocardial I/R injury in db/db mice and to investigate the underlying mechanisms involved.

## 2. Materials and Methods

### 2.1. Animal Modeling and Grouping

Male C57BLKS/J-lepr^db^/lepr^db^ diabetic (db/db; stock number 000642) and nondiabetic C57BLKS/J-lepr^+^/lepr^+^ (BKS; stock number 000662) mice (8–10 weeks) were obtained from Jackson Laboratory (Bar Harbor, Maine, USA) and housed on a 12 : 12 h light-dark cycle at 22°C with free access to food and water. db/db mice at the age of 12–16 weeks (50–60 g) when they had developed overt diabetes were used. All experiments were performed in adherence with the National Institutes of Health Guidelines on the Use of Laboratory Animals and were approved by the Fourth Military Medical University Committee on Animal Care (ID 2013052).

db/db mice were randomly allocated into the following groups with *n* = 20 each: (1) BKS + sham (sham); (2) db/db + sham (db/db); (3) db/db + I/R (I/R); (4) db/db + ZP2495 + I/R (ZP2495); (5) db/db + glucagon + I/R (glucagon); and (6) db/db + ZP131 + I/R (ZP131). The ZP2495 group was subcutaneously injected daily with ZP2495 at 70 nmol/kg of body weight for 4 weeks. The glucagon group received subcutaneous injections of glucagon at 70 nmol/kg of body weight daily for 4 weeks. The ZP131 group received daily subcutaneous injections of ZP131 at 70 nmol/kg of body weight for 4 weeks. The mice in the sham group were injected with saline at the same volume instead.

Mice were anesthetized with 2% isoflurane in 1.5 L/min O_2_. After exteriorizing the heart via a left thoracic incision, myocardial ischemia/reperfusion was induced by ligation of the left descending coronary artery for 30 minutes as previously described [19]. After 30 minutes of ischemia, hearts were harvested 3 hours (for TUNEL, caspase activity, and Western blot assay) or 24 hours (for echocardiographic, hemodynamic, and metabolic assessments and infarct size measurement) after the myocardium reperfusion.

### 2.2. Echocardiographic and Hemodynamic Measurements

Cardiac function was determined by echocardiography (VisualSonics, Canada) and invasive hemodynamic evaluation by a 1.4 French micromanometer (Millar Instruments, USA) at different time points after ischemia/reperfusion. Echocardiography was performed under anesthesia using a 30 MHz transducer on a Vevo 2100 ultrasound system (VisualSonics, Canada) as previously described [20].

### 2.3. ^18^F-Fluorodeoxyglucose Positron Emission Tomography/Computed Tomography to Assess Cardiac Glucose Metabolism

Positron emission tomography/computed tomography (PET/CT) scanning was performed using a Nano PET/CT (Mediso, Hungary) as described previously. Before imaging study, mice were maintained under fasting condition for 12–14 h, and fasting blood glucose levels were maintained between 6.0 and 7.5 mmol/L. Every animal was injected with 3.7 MBq (100 *μ*Ci) of ^18^F-FDG via tail vein injection. To obtain the best signal-to-background ratio, the animals were anesthetized under 2% isoflurane in 1.5 L/min O_2_ and acquired for whole body static PET/CT imaging at 30 min postadministration with 10 min acquisition time, energy window at 400–600 keV, and coincidence relation with 1 : 3. CT scan was subsequently obtained with 45 kV and 179 mA for 10 min. The PET images were reconstructed using ordered subset expectation maximization reconstruction (OSEM) with the SSRB 2D LOR algorithm with decay correction, CT imaging-based attenuation correction, and random corrections from raw framed sinograms. The voxel size of the PET image was 0.796 × 0.861 × 0.861 mm, for a total of 128 × 128 × 159 voxels. After PET-CT registration, 3-dimensional regions of interest (3D-ROI) were drawn over the heart region on whole body axial PET/CT fusion images. Relative accumulation of the radioactivity in particular regions of interest was expressed as standardized uptake value (SUV). Nano PET/CT images were analyzed using InterView™ Fusion software (Mediso, Hungary).

### 2.4. Myocardial Infarct Size Measurement

Myocardial infarct size was evaluated by Evans Blue/TTC staining as previously described [21].

### 2.5. Detection of Cardiomyocyte Apoptosis in Hearts

TUNEL staining and caspase-3 activity assay were used to determine cardiomyocyte apoptosis as previously described [22].

### 2.6. Transmission Electron Microscopy (TEM)

Ultrathin sections were observed under a TEM (JEM-2100, JEOL, Tokyo, Japan) at 60 kV. Mitochondria were imaged at ×10,000 (10 K) and ×40,000 (40 K) magnifications.

### 2.7. Measurement of Neonatal Rat Ventricular Myocyte (NRVM) Apoptosis Induced by Simulated Ischemia/Reperfusion (S/IR) Injury

Primary cultures of NRVMs were obtained from 1- or 2-day-old SD rats as described previously [23]. Media were replaced to different conditions after confluence: normal glucose medium (5.5 mmol/L), high-glucose medium (25 mmol/L), and high-glucose plus ZP2495 (10^−7^ mol/L), glucagon (10^−7^ mol/L), or ZP131 (10^−7^ mol/L). Simulated I/R (S/IR) was performed by transferring cardiomyocytes into an ischemic buffer adapted from Esumi et al. [24] containing (in mmol/L) NaCl 137, KCl 3.8, MgCl_2_ 0.49, CaCl_2_.2H_2_O 0.9, and HEPES 4 supplemented with deoxyglucose 10, sodium dithionate 0.75, KCl 12 and lactate 20, and pH 6.5, for 3 h in a humidified cell culture incubator (21% O_2_, 5% CO_2_, and 94% N_2_, 37°C). After simulated ischemia, cells were transferred to DMEM for 3 h in a humidified cell culture incubator (21% O_2_, 5% CO_2_, and 74% N_2_, 37°C) to simulate reperfusion.

NRVM apoptosis was determined using an Annexin V-FITC/PI Kit (Merck, USA) as previously described [25].

### 2.8. Measurement of Mitochondrial Membrane Potential (ΔΨm) (MMP)

The impact of ZP2495, glucagon, or ZP131 on mitochondrial ΔΨm in cardiomyocytes was determined by sensitive and relatively mitochondrion-specific lipophilic cationic probe fluorochrome JC-1 as previously described [26]. Cardiomyocytes were incubated with JC-1 Mitochondrial Potential Sensors (Invitrogen, USA), and images were visualized and photographed with confocal microscopy (Olympus, Japan). In order to measure the internalization of JC-1, cardiomyocytes were stained with the JC-1 Assay Kit for flow cytometry (Mitoprobe, Invitrogen, USA) according to the manufacturer's protocol and analyzed by subsequent flow cytometry (BD Bioscience, USA). To exclude cell debris and doublets, cells were gated the same number in each group, 1 × 10^4^ gated events per sample were collected from three to four independent samples per treatment condition, and the experiments were repeated at least three times. The presented data are normalized to the control group for the treated group (100%).

### 2.9. Measurement of Respiration of Mouse Cardiomyocyte

Respiration was assayed in freshly isolated mitochondria with a high-throughput-automated 96-well extracellular flux analyzer (XF96; Seahorse Bioscience, USA) as previously described [26].

### 2.10. Assessment of Mitochondrial Cytochrome c Release and ATP Content

To evaluate the subcellular localization of cytochrome c, we used confocal imaging of cells double labeled with Mitotraker Red CMX Ros (Molecular Probes, USA) and cytochrome c antibody (Cell Signaling Technology, USA). The ATP content of the myocardium was measured using an ATP bioluminescent assay kit (Beyotime, China).

### 2.11. Detection of Reactive Oxygen Species (ROS) by Mitochondria

ROS production by the mitochondria was assessed by using Mito SOX Red (Invitrogen, USA) according to product protocol. Briefly, slides were labeled with Mito SOX Red (300 nM) and Mito Tracker green (100 nM) for 20 min at 37°C, then observed by confocal microscopy (FV1000, Olympus) with the excitation wavelength at 488 and 543 nm.

### 2.12. Western Blot Assay

Cardiac tissues from the area at risk (AAR) were lysed, sonicated, and centrifuged. Western blotting was performed following standard protocol. The following primary antibodies were used: anti-phospho-FoxO3a (Thr32) (rabbit polyclonal IgG, Abcam, 1 : 100); anti-phospho-FoxO3a (Ser413) (rabbit polyclonal IgG, Cell Signaling Technology, 1 : 1000); anti-FoxO3a (rabbit polyclonal IgG, Cell Signaling Technology, 1 : 1000); anti-phospho-Akt (Ser473) (rabbit polyclonal IgG, Cell Signaling Technology, 1 : 1000); anti-phospho-Akt (Thr308) (rabbit polyclonal IgG, Cell Signaling Technology, 1 : 1000); anti-Akt (rabbit polyclonal IgG, Cell Signaling Technology, 1 : 1000); anti-AMPK (rabbit polyclonal IgG, Cell Signaling Technology, 1 : 1000); anti-phospho-AMPK (Thr172) (rabbit polyclonal IgG, Cell Signaling Technology, 1 : 1000); anti-caspase-3 (rabbit polyclonal IgG, Merck Millipore, 1 : 200); anti-cleaved caspase-3 (rabbit polyclonal IgG, Merck Millipore, 1 : 200); anti-Bim (rabbit polyclonal IgG, Abcam, 1 : 1000); anti-Bax (rabbit polyclonal IgG, Abcam, 1 : 1000); anti-Bcl-2 (rabbit monoclonal IgG, Abcam, 1 : 500); anti-Bad (rabbit polyclonal IgG, Cell Signaling Technology, 1 : 1000); anti-phospho-Bad (rabbit polyclonal IgG, Cell Signaling Technology, 1 : 1000); anti-MnSOD (rabbit polyclonal IgG, Abcam, 1 : 5000); anti-Catalase (rabbit polyclonal IgG, Abcam, 1 : 2000); anti-Sirt1 (rabbit polyclonal IgG, Abcam, 1 : 1000); anti-PGC-1*α* (rabbit polyclonal IgG, Abcam, 1 : 1000); acetylated-lysine (rabbit polyclonal IgG, Cell Signaling Technology, 1 : 1000,); anti-Nrf-1 (rabbit monoclonal IgG, Abcam, 1 : 5000); anti-Tfam (rabbit polyclonal IgG, Abcam, 1 : 2000); and *β*-actin (rabbit polyclonal IgG, Santa Cruz Biotechnology, 1 : 1000). The details are described previously [25].

### 2.13. Statistics Analysis

All data were expressed as mean ± SD and were analyzed using ANOVA, followed by a Bonferroni correction for post hoc *t*-test (with exception of Western blot). Western blot densities were analyzed with the Kruskal-Wallis test, followed by Dunn's post hoc test. A value of *P* < 0.05 was considered to be statistically significant. All statistical tests were performed using SPSS software package version 14.0 (SPSS, Chicago, IL, USA).

## 3. Results

### 3.1. Coagonist Treatment Improved Cardiac Function of db/db Mice after Myocardial I/R Injury

**Figure 1:**
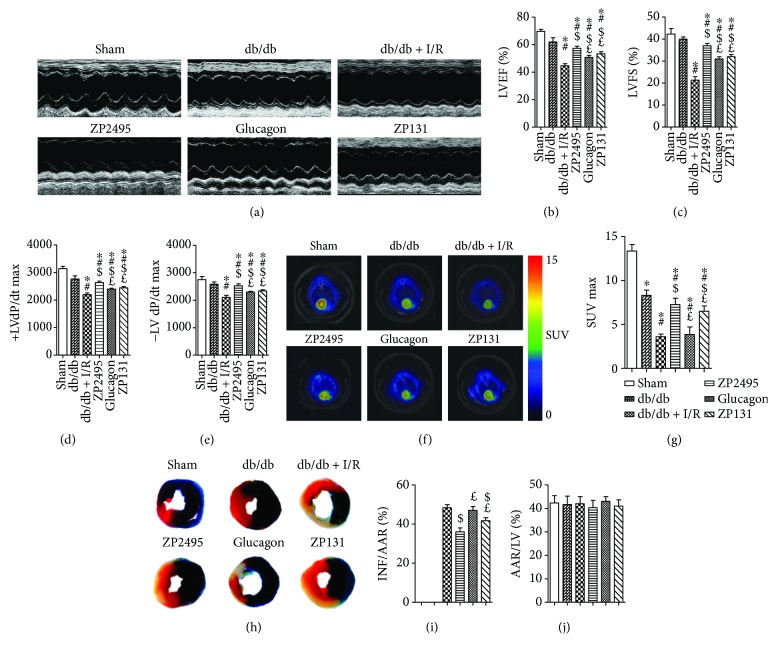
Effects of ZP2495, glucagon, and ZP131 on cardiac function and hemodynamic and cardiac glucose metabolism in db/db mice after I/R injury. (a) Representative M-mode echocardiography images. (b) Measurement of left ventricular ejection fraction (LVEF). (c) Measurement of left ventricular fractional shortening (LVFS). (d, e) Measurement of maximal velocity of pressure development and decline (±dP/dt). (f) Representative PET/CT scan images from each group. Higher glucose uptake level is evidenced by an increase in the intensity of red color. (g) Quantification of accumulated ^18^F-fluorodeoxyglucose (^18^F-FDG) in the heart. Relative accumulation of the radioactivity in particular regions of interest was expressed as standardized uptake value (SUV). (h) Myocardial infarct size was assessed by Evans blue/2,3,5-triphenyl-2H-tetrazolium chloride (TTC) double staining. Evans blue stained areas (black) indicated nonischemic/reperfused area. TTC-stained areas (red staining) indicated ischemic but viable tissue. Evans blue/TTC staining negative areas indicated infarcted myocardium. (i) Summary of infarct area (INF) per area at risk (AAR). (j) Summary of AAR per left ventricle (LV). Sham: BKS + sham group; db/db: db/db + sham group; I/R: db/db + I/R group; ZP2495: db/db + I/R + ZP2495 group; glucagon: db/db + I/R + glucagon group; and ZP131: db/db + I/R + ZP131 group. Presented values are mean ± SEM. ^∗^*P* < 0.05 versus the sham group; ^#^*P* < 0.05 versus the db/db group; ^$^*P* < 0.05 versus the db/db + I/R group; and ^£^*P* < 0.05 versus the db/db + I/R + ZP2495 group.

LVEF and LVFS were enhanced significantly in the glucagon or ZP131 group compared with the db/db + I/R group. ZP2495 enhanced LVEF and LVFS more significantly as compared with that of the glucagon or ZP131 group (Figures 1(a)–1(c)). Maximal velocity of pressure development and decline (±LV dP/dt max) also revealed a significant improvement in response to glucagon or ZP131 pretreatment in diabetic mice which underwent cardiac I/R injury. The ±LV dP/dt max was significantly higher in the ZP2495 group compared with the glucagon or ZP131 group (Figures 1(d) and 1(e)). Moreover, administration of glucagon or ZP131 reduced infarct size after cardiac I/R injury in db/db mice. The mice treated with ZP2495 exhibited much greater decline of infarct size as compared with the glucagon or ZP131 group. There was no significant difference in the area at risk (AAR) between groups (Figures 1(h)–1(j)).

### 3.2. Regulation of Cardiac Glucose Metabolism Prevented from Cardiac I/R Injury in db/db Mice

To examine the role of cardiac glucose metabolism after ZP2495, glucagon, or ZP131 treatment in db/db mice subjected to I/R, ^18^F-FDG was used to analyze the level of myocardial glucose uptake. Our data revealed that diabetic mice which underwent cardiac I/R injury had defective ^18^F-FDG uptake in the heart, which was significantly improved by glucagon or ZP131 treatment. Moreover, ^18^F-FDG uptake was significantly higher in the ZP2495 group as compared with the glucagon or ZP131 group (Figures 1(f) and 1(g)).

### 3.3. Coagonist Exerted Antiapoptotic Effect on Cardiomyocyte in db/db Mice after Cardiac I/R Injury

**Figure 2:**
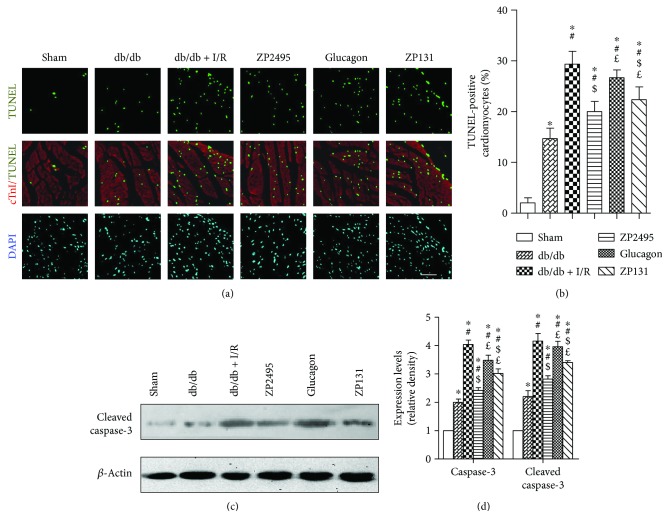
ZP2495 exerted antiapoptotic effect on cardiomyocyte of db/db mice. (a) Representative photomicrograph shows TUNEL-positive cardiomyocyte ratio, which was significantly decreased in the ZP2495 group. Apoptotic nuclei were identified as TUNEL positive (green). Myocardium was stained using a monoclonal antibody against troponin I (cTnI) (red) and total nuclei by DAPI counterstaining (blue) (scale bar = 100 *μ*m). (b) Quantitative analysis of apoptotic nuclei. Apoptotic index was termed as the percentage of apoptotic cells. (c) Representative blots of caspase-3 and cleaved caspase-3 in cardiomyocyte of db/db mice subjected to I/R. (d) Semiquantitative analysis of the Western blots. Sham: BKS + sham group; db/db: db/db + sham group; I/R: db/db + I/R group; ZP2495: db/db + I/R + ZP2495 group; glucagon: db/db + I/R + glucagon group; and ZP131: db/db + I/R + ZP131 group. Presented values are mean ± SEM. ^∗^*P* < 0.05 versus the sham group; ^#^*P* < 0.05 versus the db/db group; ^$^*P* < 0.05 versus the db/db + I/R group; and ^£^*P* < 0.05 versus the db/db + I/R + ZP2495 group.

Apoptotic cardiomyocytes labeled by TUNEL positivity were more frequently observed in the db/db + I/R group, glucagon group, and ZP131group compared with the ZP2495 group (Figure 2(a)). Quantitative analyses demonstrated that the percentage of TUNEL-positive cardiomyocytes was significantly less in the ZP2495 group compared with the db/db + I/R group or the glucagon group. However, there was no significant difference between the percentages of TUNEL-positive cardiomyocytes in the ZP2495 group and ZP131group (Figure 2(b)). Consistent with its antiapoptotic effect, ZP2495 attenuated the expression of the cleaved, activated forms of caspase-3 (Figures 2(c) and 2(d)).

### 3.4. Antiapoptotic Effect of ZP2495 on High-Glucose-Induced NRVMs under SI/R Injury

**Figure 3:**
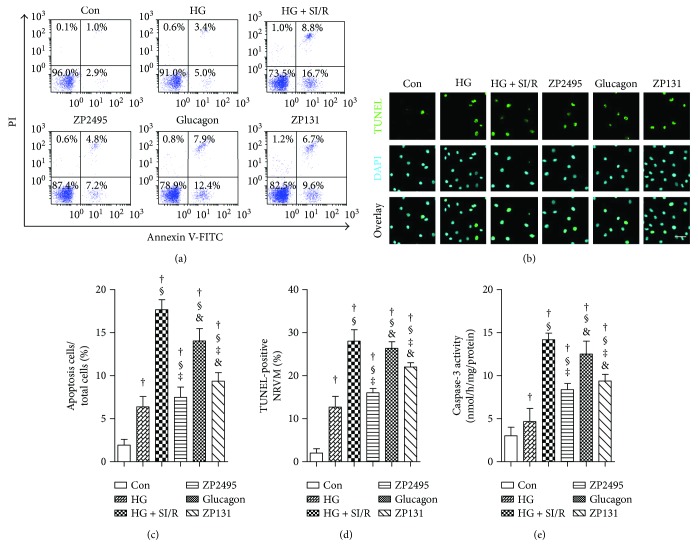
ZP2495 exerted antiapoptotic effect on high-glucose-induced NRVMs after SI/R injury. (a) Apoptosis of the NRVMs determined by annexin V/propidium iodide (PI) double staining and flow cytometry. Region Q2 late apoptotic cells, region Q3 vital cells, and region Q4 early apoptotic cells. (b) Representative images of immunostaining for apoptotic (TUNEL) cells (scale bar = 100 *μ*m). (c) Quantification of apoptosis cell by flow cytometry and TUNEL staining. (d) Quantification of apoptosis cell by TUNEL staining. (e) Activities of caspase-3. Con: normal glucose medium group; HG: high-glucose medium group; HG + SI/R: HG + SI/R group; ZP2495: HG + SI/R + ZP2495 group; glucagon: HG + SI/R + glucagon group; and ZP131: HG + SI/R + ZP131 group. Presented values are mean ± SEM. †*P* < 0.05 versus the control group; ^§^*P* < 0.05 versus the HG group; ^‡^*P* < 0.05 versus the HG + SI/R group; and ^&^*P* < 0.05 versus the HG + SI/R + ZP2495 group.

To analyze the antiapoptotic effect of ZP2495 on high-glucose-induced NRVMs after SI/R injury, we performed flow cytometry, TUNEL, and caspase-3 activity assays. Representative flow cytometry results (Figure 3(a)) indicated that high-glucose and SI/R injury significantly increased the percentage of apoptotic NRVMs. ZP2495 treatment decreased high-glucose and SI/R-induced apoptosis of NRVMs. As shown in Figures 3(b)–3(e), the antiapoptotic effect of ZP2495 on NRVMs was also evidenced by increased TUNEL (green) staining of cells (Figures 3(b)–3(d)) and upregulation of caspase-3 enzymatic activities (Figure 3(e)).

### 3.5. Antioxidation Effect of ZP2495 on Cardiomyocyte in db/db Mice under Cardiac I/R Injury via a Akt/FoxO3a-Dependent Mechanism

**Figure 4:**
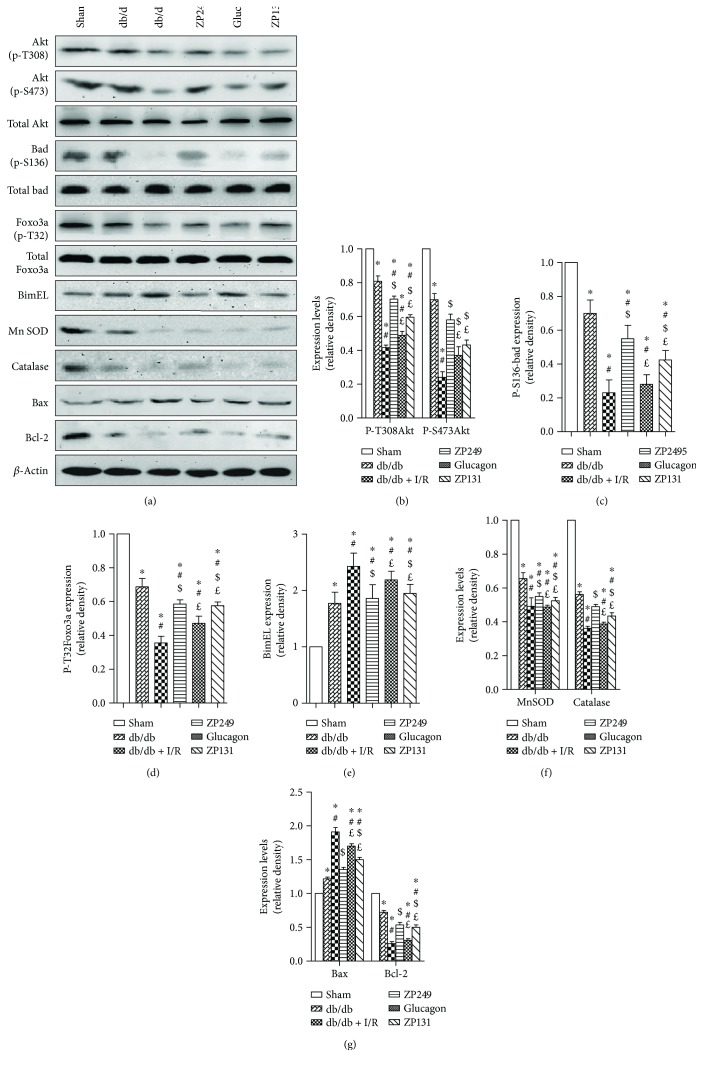
ZP2495 regulated Akt, FoxO3a, and apoptosis-related protein expression. (a) Representative blots of p-Akt, Akt, p-Bad, Bad, p-FoxO3a, FoxO3a, BimEL, MnSOD, catalase, Bax, and Bcl-2 in cardiomyocyte of db/db mice subjected to I/R. (b–g) Semiquantitative analysis of the Western blots. Sham: BKS + sham group; db/db: db/db + sham group; I/R: db/db + I/R group; ZP2495: db/db + I/R + ZP2495 group; glucagon: db/db + I/R + glucagon group; and ZP131: db/db + I/R + ZP131 group. Presented values are mean ± SEM. ^∗^*P* < 0.05 versus the sham group; ^#^*P* < 0.05 versus the db/db group; ^$^*P* < 0.05 versus the db/db + I/R group; and ^£^*P* < 0.05 versus the db/db + I/R + ZP2495 group.

To further understand the mechanism of the protective effect of ZP2495, we examined the expressions of Akt, FoxO3a, and apoptosis-related proteins using Western blot (Figure 4). ZP2495 administration increased the levels of phospho-Akt in myocardial tissue of db/db mice after cardiac I/R injury (Figures 4(a) and 4(b)). Consistently, our data further revealed reduction of the Akt-engaged phosphorylation of FoxO3 at Thr32 following ZP2495 treatment. Diabetes with cardiac I/R injury decreased FoxO3a phosphorylation at Thr32 (Figures 4(a) and 4(d)). To further examine FoxO3a activation, we measured the expression levels of its apoptosis-related downstream targets, including proapoptotic proteins BimEL and Bax, as well as antiapoptotic protein Bcl-2. The representative Western blot results demonstrated that diabetes with cardiac I/R injury increased the expressions of BimEL (Figure 4(e)) and Bax and decreased the levels of Bcl-2. ZP2495 treatment balanced the expression between pro- and antiapoptotic proteins (Figures 4(a) and 4(g)). As demonstrated in Figures 4(a) and 4(f), diabetes and I/R injury could moderately induce myocardial mitochondrial antioxidative enzyme expressions including MnSOD and catalase. ZP2495 upregulated the expression of these enzymes.

### 3.6. Coagonist Treatment Improved Mitochondrial Ultrastructural Morphology in Myocardium of db/db Mice Subjected to Cardiac I/R Injury

**Figure 5:**
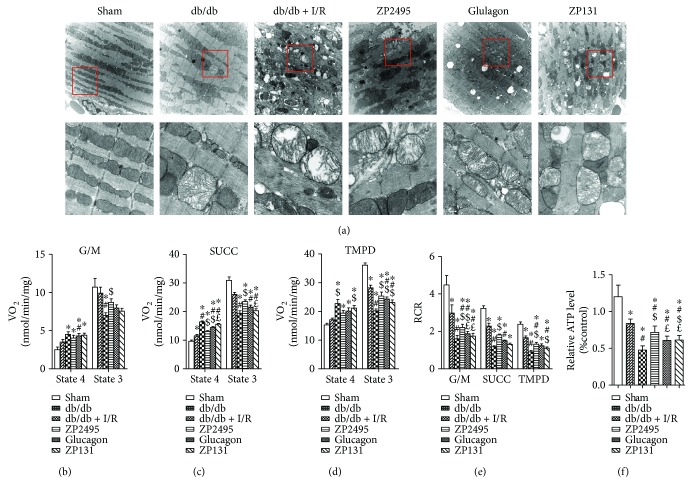
ZP2495 improved mitochondrial function and biogenesis in the myocardium of db/db mice subjected to I/R. (a) Representative transmission electron micrographs of mitochondria in the LV myocardium. Original magnification ×10,000 (scale bar = 1 *μ*m) (A1) and ×40,000 (scale bar = 250 nm) (A2). (b–d) Respiration in freshly isolated heart mitochondria from each group mice was analyzed with a SeaHorse XF96 analyzer. Mitochondria were assayed under state 4, that is, substrate but no ADP (sometimes also referred to as state 2), and state 3, that is, substrate and ADP present respiration (VO_2_) with substrates from complex I (5 mmol/L G/M) (b), complex II (5 mmol/L Succ and 1 mmol/L rotenone) (c), and complex IV (0.5 mmol/L N,N,N9,N9-tetramethyl-p-phenylenediamine (TMPD) and 3 mmol/L sodium ascorbate) (d). State 3 was induced with 1 mmol/L ADP in all cases. (e) The RCR was determined as the ratio of state 3 to state 4. The bars plotted correspond to *n* = 8 replicates from two experiments. (f) Relative ATP level change in different treatments. Sham: BKS + sham group; db/db: db/db + sham group; I/R: db/db + I/R group; ZP2495: db/db + I/R + ZP2495 group; glucagon: db/db + I/R + glucagon group; and ZP131: db/db + I/R + ZP131 group. Presented values are mean ± SEM. ^∗^*P* < 0.05 versus the sham group; ^#^*P* < 0.05 versus the db/db group; ^$^*P* < 0.05 versus the db/db + I/R group; and ^£^*P* < 0.05 versus the db/db + I/R + ZP2495 group.

Alterations in mitochondrial ultrastructure were observed by TEM. As shown in Figure 5(a), in the db/db + I/R group, most of the mitochondria from the peri-infarct zone presented significant disorders, including abnormal cristae or areas of the matrix. In some mitochondria, the cristae and matrix were cleared out, resulting in vacuoles; some mitochondria were swelling and presented with crista disorientation and breakage. The derangement in the ultrastructural morphology of mitochondria was improved in the ZP2495 treatment group, as evidenced by normalized crista density and architecture. Most of the mitochondria in this group presented sharply defined cristae. The number of swelling mitochondria was less, and no obvious vacuoles could be found in the ZP2495 treatment group.

### 3.7. Coagonist Treatment Improved Mitochondrial Energetic Transition in Myocardium of db/db Mice Subjected to Cardiac I/R Injury

We measured mitochondrial respiration, coupling of oxidative phosphorylation (OxPhos), and the relative ATP level in isolated mitochondria from hearts of all groups. Mitochondrial respiration was first quantified in the presence of substrates of complex I, II, and IV and the coupling of OxPhos under each condition (Figures 5(b)–5(d)). The basic bioenergetic behavior of mitochondria from hearts of the ZP2495 treatment group was noticeably improved. The RCR (state 3/4), a measure of the degree of coupling of OxPhos, was markedly decreased at the level of the three main respiratory complexes in mitochondria of the db/db and db/db + I/R groups (Figure 5(e)). In these two groups of mitochondria, a trend to higher values of state 4 respiration was evident in complex I, II, and IV (Figures 5(b)–5(d)). Likewise, the relative ATP levels in the db/db and db/db + I/R groups, the myocardia were also significantly decreased, and ZP2495 treatment preserved the reduction (Figure 5(f)).

### 3.8. Coagonist Treatment Prevented Mitochondrial Depolarization and Reduced Cytochrome c Release and Mitochondria ROS Generation in High-Glucose-Induced NRVMs after SI/R Injury

**Figure 6:**
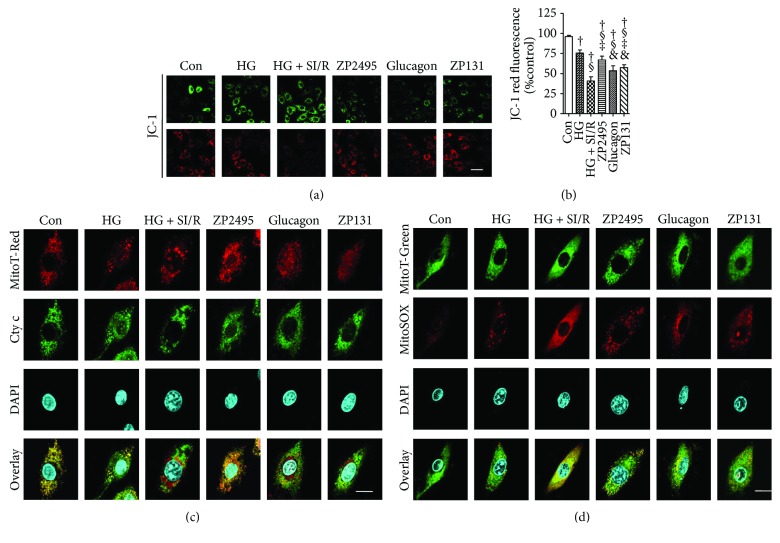
ZP2495 prevented high-glucose and SI/R-induced mitochondrial depolarization and decreased cytochrome c release and reactive oxygen species (ROS) generation from mitochondria in NRVMs. (a) Mitochondrial membrane potential (MMP) was analyzed by JC-1 fluorescence. Upper panels of green fluorescence indicated cellular uptake of JC-1, while lower panels of red fluorescence suggested depict intact mitochondria. HG and SI/R-treated NRVMs showed significantly reduced red fluorescence as compared with the control group, whereas ZP2495 treatment prevented the breakdown of the MMP as indicated by preservation of the red JC-1 fluorescence (scale bar = 100 *μ*m). (b) FACS analyses of three independent experiments with 10,000 cells per treatment condition reveal a decrease of the red JC-1 fluorescence to 41% of control levels after HG and SI/R treatment, which was prevented by ZP2495. (c) Mitotracker red fluorescence (red), cytochrome c immunoreactivity (green), DAPI staining (blue), and merged images (yellow indicates sites of colocalization) in NRVMs. Note the localization of cytochrome c in mitochondria in the NRVM control cells and ZP2495-treated cells and the diffused localization of cytochrome c in the cytoplasmic compartment of NRVM cells in HG- and SI/R-treated cultures. Representative of 25–30 cells each in 3 separate experiments (scale bar = 20 *μ*m). (d) ROS stained with MitoSOX (red) were colocalized with MitoTracker (green). Representative of 25–30 cells each in 3 separate experiments (scale bar = 20 *μ*m). Con: normal glucose medium group; HG: high-glucose medium group; HG + SI/R: HG + SI/R group; ZP2495: HG + SI/R + ZP2495 group; glucagon: HG + SI/R + glucagon group; and ZP131: HG + SI/R + ZP131 group. Presented values are mean ± SEM. ^†^*P* < 0.05 versus the control group; ^§^*P* < 0.05 versus the HG group; ^‡^*P* < 0.05 versus the HG + SI/R group; ^&^*P* < 0.05 versus the HG + SI/R + ZP2495 group.

As is shown in Figure 6(a), there were stronger red fluorescent signals and weaker green fluorescent signals in the control group, indicating intact high mitochondria membrane potential. However, red fluorescent signals had almost disappeared and green fluorescent signals were greatly enhanced in the HG (high-glucose) and HG + SI/R group, suggesting typical mitochondrial membrane potential collapse. While in the ZP2495 treated group, there were more red fluorescent signals remaining, along with fewer green fluorescent signals, which indicated that ZP2495 treatment prevented mitochondrial depolarization (Figure 6(b)). In the control group, cytochrome c immunoreactivity was colocalized with mitotraker red fluorescence, indicating that the cytochrome c was confined to the mitochondria. After high-glucose and SI/R injury, cytochrome c immunoreactivity was diffusely distributed throughout the cytoplasm and an increase in mitochondria-associated cytochrome c, indicating that cytochrome c was released from mitochondria to the cytoplasm. On the contrary, fewer cells exhibited such cytochrome c release pattern in the ZP2495 treatment group (Figure 6(c)). High glucose with SI/R injury-induced mitochondrial ROS generation was assessed by MitoSox fluorescence (Figure 6(d)). Mitochondria were visualized with Mitotracker. Image merging of MitoSox with Mitotracker showed that the diminished ROS was generated from mitochondria in the ZP2495 treatment group.

### 3.9. Coagonist Increased Mitochondrial Biogenesis in Hearts of db/db Mice Subjected to I/R Injury through AMPK/FoxO3a and AMPK-SIRT1-PGC-1*α* Pathway

**Figure 7:**
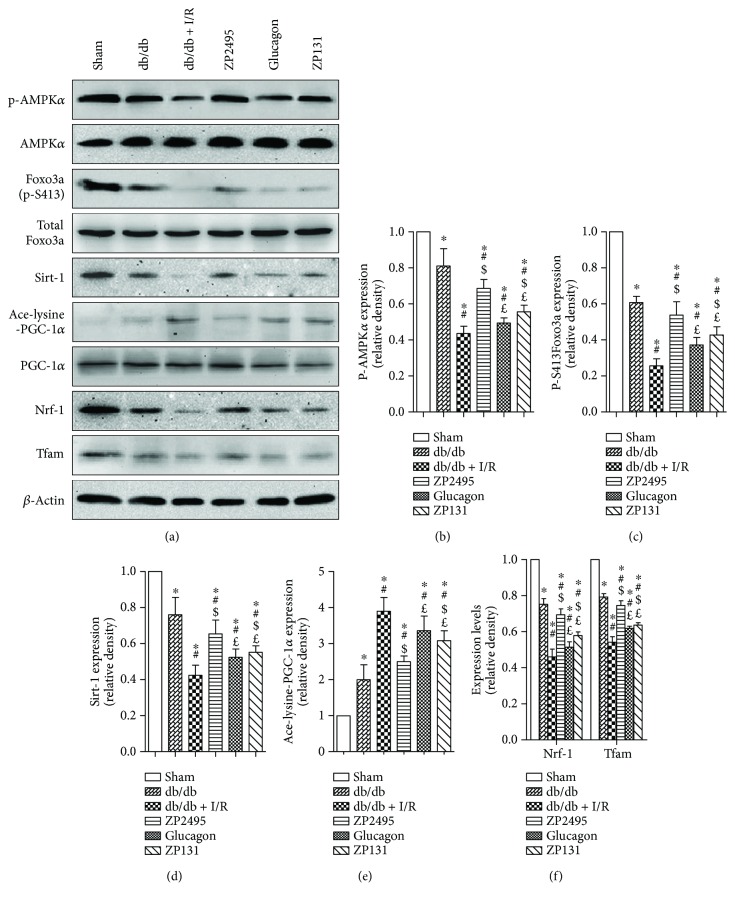
ZP2495 increased mitochondrial biogenesis in hearts of db/db mice subjected to I/R. (a) Representative blots of p-AMPK, AMPK, p-FoxO3a, FoxO3a, Sirt1, Ace-lysine-PGC-1*α*, PGC-1*α*, Nrf-1, and Tfam in cardiomyocyte of db/db mice subjected to I/R. (b–e) Semiquantitative analysis of the Western blots. Sham: BKS + sham group; db/db: db/db + sham group; I/R: db/db + I/R group; ZP2495: db/db + I/R + ZP2495 group; glucagon: db/db + I/R + glucagon group; ZP131: db/db + I/R + ZP131 group. Presented values are mean ± SEM. ^∗^*P* < 0.05 versus the sham group; ^#^*P* < 0.05 versus the db/db group; ^$^*P* < 0.05 versus the db/db + I/R group; and ^£^*P* < 0.05 versus the db/db + I/R + ZP2495 group. Data shown are representative of six independent experiments.

**Figure 8:**
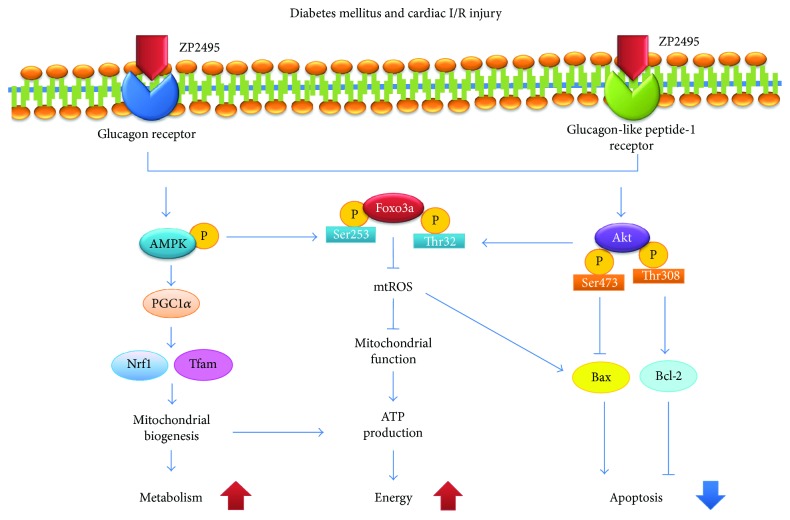
Proposed scheme for the protective effect of ZP2495 on cardiomyocyte. A summary of our current hypothesis about the mechanisms that the glucagon and GLP-1 dual-agonist (ZP2495) protects against myocardial I/R injury in db/db diabetic mice. The protective effects of ZP2495 may be associated with the activation of Akt/FoxO3 and AMPK/FoxO3a signaling pathways.

To investigate the potential signaling pathways involved in which ZP2495 improved cardiomyocyte energy metabolism after diabetes with myocardial I/R injury, we examined the level of AMPK. Our results indicated that I/R injury markedly decreased phosphorylation of AMPK in db/db mice, which was abrogated by ZP2495 (Figures 7(a) and 7(b)). Furthermore, we found reduction of AMPK-activated Foxo3 phosphorylation at the Ser413 site following ZP2495 treatment (Figures 7(a) and 7(c)). As shown in Figures 7(a)–7(e), AMPK*α* phosphorylation and SIRT1 expression level was dramatically lower and PGC-1*α* acetylation was increased in the db/db and the db/db + I/R groups. However, AMPK*α* and PGC-1*α* protein levels of cardiac tissue did not display a measurable difference between groups. ZP2495 treatment stimulated AMPK*α* phosphorylation and PGC-1*α* deacetylation without altering PGC-1*α* protein expression (Figures 7(a) and 7(e)). In the meanwhile, ZP2495 treatment significantly increased SIRT1 expression (Figures 7(a) and 7(d)). We next determined whether administration of ZP2495 could increase mitochondrial biogenesis in hearts of db/db mice subjected to I/R. ZP2495 treatment also significantly restored the protein levels of Nrf-1 and Tfam (Figures 7(a) and 7(f)). The effects of ZP2495 on mitochondrial biogenesis in db/db mice subjected to cardiac I/R injury were related to the AMPK-SIRT1-PGC-1*α* pathway (Figure 8).

## 4. Discussion

It is increasingly recognized that the occurrence of coronary artery disease contributes to an increased risk for the development of heart failure in diabetic patients. Although the pathophysiological mechanisms are certainly multifactorial, emerging evidence suggests that derangements in cardiac energy metabolism play a fundamental role in the pathogenesis of diabetic cardiomyopathy. Recent studies focusing on the mitochondria show an important role of abnormal mitochondrial function [27]. Previous studies revealed that glucagon-GLP-1 dual-agonist ZP2495 was superior compared with glucagon alone in the improvement of cardiac function in subjects with insulin resistance (IR) [28]. The data by Axelsen et al. have presented that perfusion buffer containing ZP131 failed to change cardiac function in hearts from either control or IR rats compared with vehicle-perfused hearts. In contrast, glucagon and ZP2495 perfusion improved cardiac function in control hearts. By comparison, in hearts from IR rats, however, only ZP2495 significantly increased cardiac power. Although their findings did not elucidate the protective effect of ZP2495 in the presence of diabetes, these evidences might indicate the protective role of ZP2495 in the diabetic heart because insulin resistance was usually conceived as the earlier stage of diabetes. Furthermore, ZP2495 increased glucose oxidation and glycolytic rates in IR hearts as glucagon did avoid the concomitant accumulation of AMP or ADP [28]. In line with these findings, in our current study, dual-agonist ZP2495 induced superior cardioprotection (decreased infarct size, enhanced cardiac function, and ameliorated cardiac apoptosis) in diabetic hearts subjected to myocardial I/R compared with that of ZP 131 or glucagon, which might be ascribed to its stronger capacity to upregulate cardiac glucose metabolism and mitochondrial function as compared to ZP131 or glucagon. Taken together, both our studies depicted the superiority of dual-agonist ZP2495 compared with ZP131 or glucagon in the treatment of cardiometabolic diseases with cardiac glucose metabolism defects.

The present study demonstrates that the metabolic effects observed in different regions of the ischemia-injured heart may in large part contribute to the cardioprotection observed with GLP-1 treatment [29]. However, our current study failed to analyze metabolic changes triggered by treatments in terms of ischemic versus remote regions. These changes could provide more information about a metabolic shift in viable regions of the hearts subjected to I/R injury, and we will explore this operation in our future experiments.

Myocardial I/R injury can induce additional injury to the myocardium, due to excessive oxygen free radicals, calcium overload, neutrophil infiltration, depletion of energy stores, and changes in subcellular activities including the opening of the mitochondrial permeability transition pore (MPTP) [30]. All of these changes are detrimental to ischemic cardiomyocyte and may reduce the beneficial effects of reperfusion [31]. Notably, the mechanisms activating during ischemia may lead to necrotic cell death in cardiomyocytes, while hallmarks of apoptosis mainly occur after reperfusion [32]. Accordingly, both necrotic and apoptotic cardiomyocyte cell deaths contribute to the final myocardial infarction (MI) size. Enormous preclinical and clinical studies have demonstrated that diabetes will increase the vulnerability of diabetic hearts subjected to ischemic injury and result in higher mortality [33]. Our results from the present study exhibited that ZP2495 significantly reduced infarct size after cardiac I/R injury in db/db mice, mainly by exerting an antiapoptotic effect on cardiomyocyte both *in vivo* and *in vitro*.

Cardiac mitochondria are vital organelles that supply energy to support the high ATP consumption of a beating heart [34]. The irreversible mitochondrial dysfunction is a crucial event in cardiomyocyte death during myocardial I/R injury. After reperfusion, intracellular Ca^2+^ elevation triggers mPTP opening, leading to the depletion of ATP and loss of mitochondrial integrity [35, 36]. Therefore, alleviating mitochondrial dysfunction may decrease cardiac I/R injury. Meanwhile, modulating mitochondrial function may improve insulin resistance and reduce subsequent cardiac mortality. In addition, many studies have documented signs of mitochondrial dysfunction, including impaired respiration, damaged oxidative phosphorylation, altered substrate utilization, decreased ATP production, and impaired ATP transfer [37]. In the present study, we observed both structural and functional mitochondrial impairment in the myocardium of db/db mice subjected to cardiac I/R injury. ZP2495 treatment improved mitochondrial ultrastructural morphology. These findings were in line with the alleviated functional damage observed in the mitochondria in ZP2495-treated mice.

A large accumulation of myocardial mitochondrial ROS was observed in both permanently occluded and reperfused myocardia, especially in diabetic animals, suggesting that oxidative stress in mitochondrial may contribute to cardiac dysfunction [4]. It would result in electron transport chain deterioration, mitochondrial membrane depolarization, apoptotic pathway activation, and cardiomyocyte death [4, 38]. In this study, administration of ZP2495 reduced high-glucose and SI/R injury-induced mitochondrial ROS generation, which may be responsible for preventing mitochondrial depolarization and attenuating the release of cytochrome c. These observations provide strong evidence for the notion that reduction in cardiac mitochondrial injury may represent a critical event in the cardioprotective effect of ZP2495.

Several studies have found that PGC-1*α* is important for controlling metabolic pathways in the heart during development as well as in response to physiological stressors and pathological stimuli [39, 40]. PGC-1*α* is a positive regulator of mitochondrial biogenesis and respiration and plays roles in neurodegenerative disorders and heart failure and pathological conditions that are strongly associated with mitochondrial defects [39]. Recent studies have shown that PGC-1*α* acetylation was a kind of protein modification with inactivation effects [41]. PGC-1*α* activity has also been reported to be modulated by several modifications, such as phosphorylation by AMPK or deacetylation by AMPK and sirtuin 1 (SIRT1). In the current study, we observed that ZP2495 upregulated PGC-1*α* and its downstream Nrf-1 and Tfam expressions, which was associated with an increase in mitochondrial DNA copies in db/db mouse hearts. These results indicated that ZP2495 may exhibit its effect of cardioprotection through improving mitochondrial biogenesis by activating the AMPK/PGC-1*α* pathway.

Another important observation of our study is that FoxO3a may play an important role in protecting the myocardium from injury. The FoxO transcription factors, including FoxO1, FoxO3a, FoxO4, and FoxO6, belong to the forkhead family of transcriptional regulators. FoxO transcription factors have been implicated in regulating diverse cellular functions such as differentiation, metabolism, proliferation, and apoptosis [42, 43], especially for Akt-mediated antiapoptotic properties [23]. Furthermore, FoxO3a has been considered a converging point for Akt and AMPK signaling pathways [44, 45]. AMPK phosphorylation of FoxO3a at the site of Ser413 enhances the stress resistance and longevity [46–50]. On the other hand, Akt phosphorylation of FoxO3a at Thr32 site inhibits FoxO3a transcriptional activity, promoting antiapoptosis and insulin sensitivity [51]. Therefore, Akt and AMPK signaling may elicit transcriptional regulation via FoxO3a to allow organismal adaptation for physiological or pathophysiological changes. The convergence of the two pathways at the level of FoxO3a may play a critical role in the crosstalk between Akt and AMPK. Our data revealed that the protective effect of ZP2495 was related to FoxO3a activation mediated by the PI3K/Akt and AMPK pathways. We found that ZP2495 phosphorylated FoxO3a to inhibit the transcription of proapoptotic gene Bim, which resulted in decreased expression of proapoptotic proteins such as Bim and Bax. Consequently, cytochrome c was released into the cytosol and the caspase cascade was activated. In parallel, ZP2495 increased levels of the antiapoptotic proteins Bad and Bcl-2, which alleviated the apoptosis induced by diabetes and cardiac I/R injury. Collectively, we demonstrated that ZP2495 exerted an antiapoptotic effect by inhibiting ROS production, cytochrome c release, and caspase-3 activation through Akt/FoxO3a and AMPK/FoxO3a pathways. In the present study, myocardial I/R injury induced loss of phosphorylation of Akt and AMPK in db/db mice. These data suggested a potential role of Akt-FoxO3a and AMPK-FoxO3a in cardiac I/R injury and diabetes-induced mitochondrial dysfunction and apoptosis. Our data revealed a novel mechanism whereby ZP2495 exerted its protective effect by activating FoxO3a through the Akt and AMPK signaling pathways.

### 4.1. Limitations

Other members of the RISK pathway may also participate in the link between ZP2495 and its cardiac protection effects. In order to further demonstrate the efficacy of ZP2495 administration on cardiomyocyte mitochondrial biogenesis and function, AMPK-related pathways should be investigated in future studies. More studies should be performed to figure out whether ZP2495 activates Akt/FoxO3a and AMPK/FoxO3a pathways by different mechanisms in diabetic mice or nondiabetic mice which underwent cardiac I/R injury.

## 5. Conclusion

In conclusion, we provided evidences that glucagon and GLP-1 dual-agonist ZP2495 protected against myocardial I/R injury in db/db mice, which may be attributed to the improvement of cardiac mitochondrial function and energy metabolism. These protective effects of ZP2495 might be associated with Akt/FoxO3a and AMPK/FoxO3a signaling pathways.

## References

[1] ACCORD Study Group, Gerstein H. C., Miller M. E. (2011). Long-term effects of intensive glucose lowering on cardiovascular outcomes. *The New England Journal of Medicine*.

[2] Coch R. W., Green J. B. (2016). Current cardiovascular outcomes trials in type 2 diabetes: perspectives and insight. *Nutrition, Metabolism & Cardiovascular Diseases*.

[3] Sun D., Narsinh K., Wang H. (2013). Effect of autologous bone marrow mononuclear cells transplantation in diabetic patients with ST-segment elevation myocardial infarction. *International Journal of Cardiology*.

[4] Koka S., Das A., Salloum F. N., Kukreja R. C. (2013). Phosphodiesterase-5 inhibitor tadalafil attenuates oxidative stress and protects against myocardial ischemia/reperfusion injury in type 2 diabetic mice. *Free Radical Biology & Medicine*.

[5] Drucker D. J., Sherman S. I., Gorelick F. S., Bergenstal R. M., Sherwin R. S., Buse J. B. (2010). Incretin-based therapies for the treatment of type 2 diabetes: evaluation of the risks and benefits. *Diabetes Care*.

[6] Drucker D. J. (2016). The cardiovascular biology of glucagon-like peptide-1. *Cell Metabolism*.

[7] Wallner M., Kolesnik E., Ablasser K. (2015). Exenatide exerts a PKA-dependent positive inotropic effect in human atrial myocardium: GLP-1R mediated effects in human myocardium. *Journal of Molecular and Cellular Cardiology*.

[8] Sun Z., Tong G., Kim T. H. (2015). PEGylated exendin-4, a modified GLP-1 analog exhibits more potent cardioprotection than its unmodified parent molecule on a dose to dose basis in a murine model of myocardial infarction. *Theranostics*.

[9] Marso S. P., Daniels G. H., Brown-Frandsen K. (2016). Liraglutide and cardiovascular outcomes in type 2 diabetes. *The New England Journal of Medicine*.

[10] Farah A., Tuttle R. (1960). Studies on the pharmacology of glucagon. *The Journal of Pharmacology and Experimental Therapeutics*.

[11] Mery P. F., Brechler V., Pavoine C., Pecker F., Fischmeister R. (1990). Glucagon stimulates the cardiac Ca^2+^ current by activation of adenylyl cyclase and inhibition of phosphodiesterase. *Nature*.

[12] Mayer S. E., Namm D. H., Rice L. (1970). Effect of glucagon on cyclic 3′,5′-AMP, phosphorylase activity and contractility of heart muscle of the rat. *Circulation Research*.

[13] Stanley W. C., Lopaschuk G. D., McCormack J. G. (1997). Regulation of energy substrate metabolism in the diabetic heart. *Cardiovascular Research*.

[14] Henderson S. J., Konkar A., Hornigold D. C. (2016). Robust anti-obesity and metabolic effects of a dual GLP-1/glucagon receptor peptide agonist in rodents and non-human primates. *Diabetes, Obesity and Metabolism*.

[15] Lopaschuk G. D., Wall S. R., Olley P. M., Davies N. J. (1988). Etomoxir, a carnitine palmitoyltransferase I inhibitor, protects hearts from fatty acid-induced ischemic injury independent of changes in long chain acylcarnitine. *Circulation Research*.

[16] Heather L. C., Pates K. M., Atherton H. J. (2013). Differential translocation of the fatty acid transporter, FAT/CD36, and the glucose transporter, GLUT4, coordinates changes in cardiac substrate metabolism during ischemia and reperfusion. *Circulation: Heart Failure*.

[17] Lopaschuk G. D., Wambolt R. B., Barr R. L. (1993). An imbalance between glycolysis and glucose oxidation is a possible explanation for the detrimental effects of high levels of fatty acids during aerobic reperfusion of ischemic hearts. *The Journal of Pharmacology and Experimental Therapeutics*.

[18] Liu B., Clanachan A. S., Schulz R., Lopaschuk G. D. (1996). Cardiac efficiency is improved after ischemia by altering both the source and fate of protons. *Circulation Research*.

[19] Gao E., Lei Y. H., Shang X. (2010). A novel and efficient model of coronary artery ligation and myocardial infarction in the mouse. *Circulation Research*.

[20] Zhang M., Sun D., Li S. (2015). Lin28a protects against cardiac ischaemia/reperfusion injury in diabetic mice through the insulin-PI3K-mTOR pathway. *Journal of Cellular and Molecular Medicine*.

[21] Sun D., Huang J., Zhang Z. (2012). Luteolin limits infarct size and improves cardiac function after myocardium ischemia/reperfusion injury in diabetic rats. *PLoS One*.

[22] Gao H. K., Yin Z., Zhou N., Feng X. Y., Gao F., Wang H. C. (2008). Glycogen synthase kinase 3 inhibition protects the heart from acute ischemia-reperfusion injury via inhibition of inflammation and apoptosis. *Journal of Cardiovascular Pharmacology*.

[23] Yan W., Zhang H., Liu P. (2013). Impaired mitochondrial biogenesis due to dysfunctional adiponectin-AMPK-PGC-1*α* signaling contributing to increased vulnerability in diabetic heart. *Basic Research in Cardiology*.

[24] Esumi K., Nishida M., Shaw D., Smith T. W., Marsh J. D. (1991). NADH measurements in adult rat myocytes during simulated ischemia. *The American Journal of Physiology*.

[25] Zhang Z., Li S., Cui M. (2013). Rosuvastatin enhances the therapeutic efficacy of adipose-derived mesenchymal stem cells for myocardial infarction via PI3K/Akt and MEK/ERK pathways. *Basic Research in Cardiology*.

[26] Li N., Yuan Y., Li S. (2016). PDE5 inhibitors protect against post-infarction heart failure. *Frontiers in Bioscience*.

[27] Boudina S., Sena S., Theobald H. (2007). Mitochondrial energetics in the heart in obesity-related diabetes: direct evidence for increased uncoupled respiration and activation of uncoupling proteins. *Diabetes*.

[28] Axelsen L. N., Keung W., Pedersen H. D. (2012). Glucagon and a glucagon-GLP-1 dual-agonist increases cardiac performance with different metabolic effects in insulin-resistant hearts. *British Journal of Pharmacology*.

[29] Aravindhan K., Bao W., Harpel M. R., Willette R. N., Lepore J. J., Jucker B. M. (2015). Cardioprotection resulting from glucagon-like peptide-1 administration involves shifting metabolic substrate utilization to increase energy efficiency in the rat heart. *PLoS One*.

[30] Rosano G., Fini M., Caminiti G., Barbaro G. (2008). Cardiac metabolism in myocardial ischemia. *Current Pharmaceutical Design*.

[31] Ibanez B., Fuster V., Jimenez-Borreguero J., Badimon J. J. (2011). Lethal myocardial reperfusion injury: a necessary evil?. *International Journal of Cardiology*.

[32] Gottlieb R. A., Burleson K. O., Kloner R. A., Babior B. M., Engler R. L. (1994). Reperfusion injury induces apoptosis in rabbit cardiomyocytes. *The Journal of Clinical Investigation*.

[33] Tao L., Gao E., Jiao X. (2007). Adiponectin cardioprotection after myocardial ischemia/reperfusion involves the reduction of oxidative/nitrative stress. *Circulation*.

[34] Lemieux H., Semsroth S., Antretter H., Hofer D., Gnaiger E. (2011). Mitochondrial respiratory control and early defects of oxidative phosphorylation in the failing human heart. *The International Journal of Biochemistry & Cell Biology*.

[35] Miller B. A., Hoffman N. E., Merali S. (2014). TRPM2 channels protect against cardiac ischemia-reperfusion injury: role of mitochondria. *The Journal of Biological Chemistry*.

[36] Woodman O. L., Long R., Pons S., Eychenne N., Berdeaux A., Morin D. (2014). The cardioprotectant 3′,4′-dihydroxyflavonol inhibits opening of the mitochondrial permeability transition pore after myocardial ischemia and reperfusion in rats. *Pharmacological Research*.

[37] Arkat S., Umbarkar P., Singh S., Sitasawad S. L. (2016). Mitochondrial Peroxiredoxin-3 protects against hyperglycemia induced myocardial damage in diabetic cardiomyopathy. *Free Radical Biology & Medicine*.

[38] Loor G., Kondapalli J., Iwase H. (2011). Mitochondrial oxidant stress triggers cell death in simulated ischemia–reperfusion. *Biochimica et Biophysica Acta (BBA) - Molecular Cell Research*.

[39] Finck B. N., Kelly D. P. (2007). Peroxisome proliferator–activated receptor *γ* coactivator-1 (PGC-1) regulatory cascade in cardiac physiology and disease. *Circulation*.

[40] Huang C. C., Wang T., Tung Y. T., Lin W. T. (2016). Effect of exercise training on skeletal muscle SIRT1 and PGC-1*α* expression levels in rats of different age. *International Journal of Medical Sciences*.

[41] Jeninga E. H., Schoonjans K., Auwerx J. (2010). Reversible acetylation of PGC-1: connecting energy sensors and effectors to guarantee metabolic flexibility. *Oncogene*.

[42] van der Horst A., Burgering B. M. T. (2007). Stressing the role of FoxO proteins in lifespan and disease. *Nature Reviews Molecular Cell Biology*.

[43] Wang Y., Zhou Y., Graves D. T. (2014). FOXO transcription factors: their clinical significance and regulation. *BioMed Research International*.

[44] Greer E. L., Oskoui P. R., Banko M. R. (2007). The energy sensor AMP-activated protein kinase directly regulates the mammalian FOXO3 transcription factor. *The Journal of Biological Chemistry*.

[45] Paula-Gomes S., Gonçalves D., Baviera A., Zanon N., Navegantes L., Kettelhut I. (2013). Insulin suppresses atrophy- and autophagy-related genes in heart tissue and cardiomyocytes through AKT/FOXO signaling. *Hormone and Metabolic Research*.

[46] Greer E. L., Dowlatshahi D., Banko M. R. (2007). An AMPK-FOXO pathway mediates longevity induced by a novel method of dietary restriction in *C. elegans*. *Current Biology*.

[47] Bodur C., Karakas B., Timucin A. C., Tezil T., Basaga H. (2016). AMP-activated protein kinase couples 3-bromopyruvate-induced energy depletion to apoptosis via activation of FoxO3a and upregulation of proapoptotic Bcl-2 proteins. *Molecular Carcinogenesis*.

[48] Queiroz E. A. I. F., Fortes Z. B., da Cunha M. A. A., Barbosa A. M., Khaper N., Dekker R. F. H. (2015). Antiproliferative and pro-apoptotic effects of three fungal exocellular *β*-glucans in MCF-7 breast cancer cells is mediated by oxidative stress, AMP-activated protein kinase (AMPK) and the forkhead transcription factor, FOXO3a. *The International Journal of Biochemistry & Cell Biology*.

[49] Shrestha A., Nepal S., Kim M. J. (2016). Critical role of AMPK/FoxO3A Axis in globular adiponectin-induced cell cycle arrest and apoptosis in cancer cells. *Journal of Cellular Physiology*.

[50] Xia W., Zhang F., Xie C., Jiang M., Hou M. (2015). Macrophage migration inhibitory factor confers resistance to senescence through CD74-dependent AMPK-FOXO3a signaling in mesenchymal stem cells. *Stem Cell Research & Therapy*.

[51] Ni Y. G., Wang N., Cao D. J. (2007). FoxO transcription factors activate Akt and attenuate insulin signaling in heart by inhibiting protein phosphatases. *Proceedings of the National Academy of Sciences of the United States of America*.

